# The case for viral load testing in adolescents in resource‐limited settings

**DOI:** 10.1002/jia2.25002

**Published:** 2017-11-24

**Authors:** Rebecca Marcus, Rashida A Ferrand, Katharina Kranzer, Linda‐Gail Bekker

**Affiliations:** ^1^ The Desmond Tutu HIV Centre University of Cape Town Cape Town South Africa; ^2^ Barts Health NHS Trust London UK; ^3^ Clinical Research Department London School of Hygiene and Tropical Medicine London UK; ^4^ National and Supranational Reference Laboratory Research Centre Leibnitz Borstel Germany; ^5^ Biomedical Research and Training Institute Harare Zimbabwe

**Keywords:** adolescent, viral load, HIV, resource‐limited

## Abstract

**Introduction:**

The success of HIV treatment programmes globally has resulted in children with perinatally acquired HIV reaching adolescence in large numbers. The number of adolescents living with HIV is growing further due to persisting high HIV incidence rates among adolescents in low‐ and middle‐income settings, particularly in sub‐Saharan Africa. Although expanding access to HIV viral load monitoring is necessary to achieve the 90‐90‐90 targets across the HIV care continuum, implementation is incomplete. We discuss the rationale for prioritizing viral load monitoring among adolescents and the associated challenges.

**Discussion:**

Adolescents with HIV are a complex group to treat successfully due to extensive exposure to antiretroviral therapy for those with perinatally acquired HIV and the challenges in sustained medication adherence in this age group. Given the high risk of treatment failure among adolescents and the limited drug regimens available in limited resource settings, HIV viral load monitoring in adolescents could prevent unnecessary and costly switches to second‐line therapy in virologically suppressed adolescents. Because adolescents living with HIV may be heavily treatment experienced, have suboptimal treatment adherence, or may be on second or even third‐line therapy, viral load testing would allow clinicians to make informed decisions about increased counselling and support for adolescents together with the need to maintain or switch therapeutic regimens.

**Conclusions:**

Given scarce resources, prioritization of viral load testing among groups with a high risk of virological failure may be required. Adolescents have disproportionately high rates of virological failure, and targeting this age group for viral load monitoring may provide valuable lessons to inform broader scale‐up.

## Introduction

The numbers of adolescents living with HIV has increased globally by 30% since 2005 1. The paediatric HIV epidemic is maturing, with increasing numbers of children living into adolescence and young adulthood due to the scale‐up of antiretroviral therapy (ART), and HIV‐infected children with slow disease progression presenting to health services for the first time in adolescence 2, 3. The persisting high incidence of HIV among adolescents in low and middle‐income settings (LMICs), particularly among young women and girls, is also a significant contributor to the increase in numbers of adolescents living with HIV over the past decade. HIV is the second biggest cause of death among adolescents globally, and accounts for a substantial proportion of deaths in sub‐Saharan Africa 4. Notably, HIV‐related deaths among adolescents have risen during a period in which there has been a significant decline in HIV‐related mortality among other age groups 5.

HIV viral load (VL) testing allows accurate monitoring of antiretroviral treatment and detection of virological failure. Expanding access to VL monitoring is necessary to achieve the ambitious UNAIDS treatment target that stipulates that 90% that those on ART should have durable viral suppression. Universal access to HIV VL testing has yet to be achieved however. We discuss the rationale for prioritizing VL monitoring among adolescents.

## The state of the adolescent HIV epidemic

An estimated 1.8 million adolescents between 10 and 19 years old were living with HIV in 2015, an increase of 28 per cent since 2005 6. The majority of these adolescents live in resource‐limited settings, 82% in sub‐Saharan Africa, and nearly half of the estimated numbers of adolescents living with HIV are in five countries, India, Kenya, Nigeria, South Africa and Tanzania 4, 7. This growing cohort of adolescents comprises of two groups: those with perinatally acquired infection born before widely available prevention of mother‐to‐child transmission (PMTCT) who are surviving into adolescence and young adulthood, and those with largely sexually acquired new infections 8. The numbers of children infected with HIV is declining due to the concerted global effort of PMTCT. The use of antiretroviral therapy as part of PMTCT programmes has averted 350,000 new paediatric HIV infections, with a remarkable reduction in the annual number of new infections amongst children by 70% since 2001 9.

Children with perinatally acquired HIV who would otherwise have died in infancy and early childhood are now reaching adolescence in large numbers due to earlier diagnosis, treatment initiation and the global scale‐up of antiretroviral therapy (ART) 10. At least a third of HIV‐infected infants have slow progressing disease, with a median survival of at least 16 years 11, 12. Many children infected a decade or so ago before PMTCT programmes were available therefore present to health services for the first time in adolescence 1.

While the rate of new infections among adults has stabilized in many countries, the decline in the incidence of infection among adolescents and young people has been slower. A third of new infections occur in the 15–24 year age group, with an estimated 670,000 young people in this age group newly infected with HIV in 2015, representing over 40% of new HIV infections globally 13; of these, 250,000 were adolescents aged 15–19 years old 8.

This growing cohort of adolescents presents a challenge to resource‐limited health services not used to effectively managing adolescents, a subset of whom have complex treatment histories and chronic illness 10.

## Adolescents and treatment outcomes

Adolescence is a period of physiological and psychological growth characterized by biological, sexual and identity development and increasing social autonomy 14, 15. This stage of development is a high‐risk period in terms of engagement with healthcare services, as adolescents often experience destabilizing socio‐cultural change coupled with increased risk‐taking behaviour and decreased parental support 16. Poor adherence to treatment during adolescence has been reported for many chronic conditions, including diabetes, asthma and epilepsy 16. HIV is no exception, and adolescents living with HIV are not only less likely to initiate ART than adults, but those who do start ART are less likely to remain in care and adhere to treatment 17. Intermittent medication adherence increases the risk of treatment failure and subsequent development of drug resistance 18, 19. Studies in high‐ and low‐resource settings have shown that adolescents have worse virological outcomes than other age groups 20, 21, 22. Those with chronic conditions may be more impacted by the risk‐taking behaviours that characterize adolescence than their peers without chronic medical problems in terms of impact on their health and ability to manage their illness 23, 24. Living with chronic disease can result in considerable psychological burden, involving social and educational disruption, real or perceived stigma, and the difficulties of adjusting to adult healthcare services as they transition from paediatric care 25. In addition, particularly those adolescents with perinatally acquired infection may have lost one or both parents to HIV, with significant economic, social and psychological consequences and adverse impacts on treatment access and health outcomes 26.

Both adolescents with perinatally acquired and non‐perinatally acquired HIV have varied and overlapping risk factors for treatment failure and subsequent development of drug resistance. Adolescents in both groups face the complexities of living with an oft‐stigmatized condition throughout adolescence and into adulthood with the attendant risks on adherence and retention in care.

Adolescents with perinatally acquired HIV are at high risk for treatment failure and multiclass drug resistance for several reasons. Those who have reached adolescence are likely to have had exposure to ART to prevent perinatal HIV infection or started ART early in life, and may be heavily treatment experienced 27, 28. Subtherapeutic drug concentrations caused by limited paediatric drug formulations, variable pharmacokinetics and physiological changes may increase the risk of drug‐resistant virus with subsequent virologic failure 29, 30, 31. Many perinatally infected adolescents today would have started ART early in life in a time when triple ART regimens were unavailable 1. Mono‐ or dual therapy regimens may have resulted in incomplete viral suppression and the emergence of drug‐resistant virus 32, 33, 34. In a European multicentre paediatric HIV cohort, 25% had triple‐class failure after 8 years on ART 34, and in LMICs, multiclass drug resistance of up to 90% has been demonstrated in perinatally infected children and adolescents 20, 27, 35. In the absence of VL testing, detection of treatment failure may be delayed and patients may be maintained on inadequate and subtherapeutic treatment, with subsequent increased risk of development of drug resistance 36. The consequences of drug‐resistant virus and treatment failure may have life limiting consequences in children and adolescents who will require treatment for many years longer than adults.

Adolescents with recently acquired HIV infection are also at substantial risk of treatment failure and drug resistance 37, 38. HIV acquisition in adolescence is often associated with high‐risk sexual behaviour including low rates of condom use, multiple concurrent partners, transgenerational and transactional sex 39. In sub‐Saharan Africa, adolescent girls have a four times higher prevalence of HIV than their male counterparts 4. Adolescents from key populations, including men who have sex with men (MSM), transgender people, injecting drug users and commercial sex workers have a particularly high HIV prevalence 4. Available data suggest that young MSM often have a higher risk of acquiring HIV than heterosexual young men or older MSM, with prevalence rates of between 3% and 24% reported across a diverse range of settings in 10–24 year olds 40. The risk factors and social conditions that lead to HIV acquisition in adolescence may subsequently impact on their ability to engage in care, with the attendant risks of treatment failure 41. Sexual risk behaviours and subsequent HIV risk in adolescents are also subject to stigmatization 39, 42. HIV‐related stigma in youth correlates with factors that threaten ART adherence with an increased potential for developing drug‐resistant virus 43, 44, 45. Data from the USA reports a high prevalence of genotypic and phenotypic drug resistance in newly infected youth 37, 38 and more data is emerging from LMICs on the rising prevalence of transmitted drug resistance in newly infected adolescents and adults 46, 47.

## The case for viral load monitoring for adolescents

Viral load testing is recommended by the WHO as the gold standard for monitoring HIV treatment and failure in all patients 48. In high‐income countries, HIV VL testing is routinely used for monitoring patients on ART, whereas in resource‐limited settings, clinical and immunologic (CD4 count) monitoring is largely used to diagnose treatment failure 49. It is recognized that this approach is inadequate for treatment monitoring as clinical or immunological monitoring delays recognition of virological failure with potential accumulation of drug resistance mutations and a subsequent reduction in future treatment options 50. It may also lead to unnecessary switching to second‐line therapy in those with suspected poor adherence, for example in those whose CD4 counts have not fully reconstituted since ART initiation or in those who develop an opportunistic infection but are in fact virologically suppressed 51, 52.

VL monitoring is not available in most resource‐limited settings 1, 27, 50. While all populations living with HIV would benefit from the scale‐up of VL testing, VL roll‐out could initially be targeted at certain high‐risk populations especially vulnerable to virological failure such as during adolescence, a period when maintaining sustained adherence to treatment and engagement with care is particularly challenging 13, 53. Although universal VL monitoring in LMIC may not be cost‐effective while costs of VL testing remain high, selecting key populations at risk of poor adherence might allow for more efficient use of resources by identifying those with an undetectable VL for whom less intensive differentiated care options are more appropriate, and highlighting those individuals who need more intensive support 54.

Adolescence is also a period of increased sexual risk taking with subsequent risk of both HIV acquisition and transmission and of unplanned pregnancy 55, 56. VL monitoring will allow those who not virologically suppressed to be identified, thereby allowing targeted adherence interventions to reduce onward sexual and vertical transmission. Young people begin to assert more autonomous control over their decisions during adolescence 14. Chronic illness can engender a feeling of powerlessness amongst adolescents, who may then choose to exercise control by not taking medication, or attending appointments for example 57. Using VL testing as a definitive marker of treatment outcomes may allow for active adolescent participation in tracking self‐progress.

As adolescents with perinatally acquired HIV may be heavily treatment experienced and on second or even third‐line therapy, and both those with perinatally acquired HIV and newly infected adolescents may have suboptimal treatment adherence, VL testing in this age group would allow clinicians to make informed decisions about increased counselling and support for adolescents together with the need to maintain or switch therapeutic regimens. VL monitoring accompanied by appropriate regimen change will reduce the time spent on a failing regimen avoiding the selection of resistant viral mutations 58. This is of particular importance in adolescents with perinatally acquired HIV, who often have a history of inadequate viral suppression. Even without widespread availability of genotypic resistance testing, VL monitoring would give clinicians an important decision‐making tool that will impact an adolescent's treatment options for many years.

In low‐ and middle‐income countries where limited VL testing is available, measuring VL six months after treatment initiation gives healthcare professionals the opportunity to intervene with targeted adherence and support interventions 52, 59. Risk factors such as poor adherence, female gender, high baseline VL and non‐nucleoside reverse transcriptase inhibitor (NNRTI) based‐regimes have been associated with virological failure among adolescents 27, 34. Preservation of ART regimens is important in an adolescent population who will require ART for longer than adults. Given that adolescents are at higher risk of treatment failure when compared with adults, targeting VL testing at certain adolescent subgroups at high risk of failure may increase the yield of detecting treatment failure and make regime choices more efficient.

## Opportunities for developing VL monitoring for adolescents

Adolescents are a mobile group, with migration linked to key transitions into adulthood, for example from rural to urban areas for employment or educational opportunities 60. Engaging and retaining these populations in care using traditional healthcare facilities is challenging, and innovative and adolescent‐responsive differentiated models of HIV care may improve outcomes across the HIV care continuum 61. Point‐of‐care (POC) technology may be particularly beneficial in targeting adolescents as part of a hard‐to‐reach‐group 62. Although cost of POC technology is currently prohibitive for widespread roll‐out in low‐resource settings, research and development for a semiquantitative POC VL assay is in progress 63. Near‐patient VL testing technology could be incorporated into mobile health clinics for example, which use rapid and near‐patient technologies for HIV testing and CD4 count monitoring to engage hard‐to‐reach adolescent populations in care 62. Flexible methods of VL testing including decentralized and near‐patient VL testing with rapid availability of results will allow clinicians to make prompt decisions about treatment failure or the need for regime change, and may obviate need for repeated clinic visits thereby reducing educational, employment and social disruption. Blood obtained from a finger prick with results read from a test strip, much like a POC HIV test, would remove the need for phlebotomists or centrifugation and may be more acceptable to adolescents than venesection 64. One such POC VL system has received regulatory approval in Malawi, and product approval in Uganda and Kenya, but has not been validated for paediatric or adolescent use 63.

Phased implementation of VL testing would allow logistical and technical capacity‐building for universal access in a context where introduction of routine VL monitoring for the general population living with HIV is not feasible. Initial implementation of VL assays in LMICs could prioritize groups such as adolescents who are at higher risk of treatment failure and constitute a smaller group compared to adults with HIV. Initiating VL testing in adolescents as a sub‐section of the population living with HIV could provide a platform for investigating the feasibility, cost‐effectiveness, and acceptability of VL testing in these settings, with the potential to inform wider scale implementation.

## Conclusions

The impact of global ART programme scale‐up has produced astounding gains in mortality, turning an inevitably fatal condition into a chronic disease requiring lifelong treatment with sustained adherence. The Global Plan for the eradication of vertical transmission of HIV has significantly reduced the number of children born with HIV. Early diagnosis and treatment has resulted in most of these children reaching adolescence. The global community set the goal of ending the HIV epidemic by 2030, with ambitious targets to get 90% of people with HIV tested, 90% of those tested on treatment and 90% of those on treatment virally suppressed. Yet in 2016, we saw almost 2 million new HIV infections, most of those in adolescents and young people. As universal ‘test‐and‐treat’ becomes reality, the numbers initiating ART will rise. One group who will continue to pose significant treatment challenges are adolescents either graduating from paediatric care or newly established on ART treatment who may encounter treatment fatigue with an associated risk of failure. Unlike in the early days of ART where saving lives was the primary objective, we are entering a phase where VL testing is essential in order to successfully maintain people on long‐term ART to ensure the promise of universal treatment scale‐up. VL testing scale‐up is critical in all groups, yet, focusing on adolescents for initial operational research and scale‐up will allow targeted investigation into optimum ways of integrating VL monitoring into differentiated models of care, cost‐effectiveness and evaluating how this cost can be weighed against other HIV prevention and care programme priorities for this vulnerable group.

## Competing interests

The authors do not have any competing interests to declare.

## Authors’ contributions

All authors contributed to writing and review, and all approved the final version.
